# Switching From Proximal to Distal Radial Artery Access for Coronary Chronic Total Occlusion Recanalization

**DOI:** 10.3389/fcvm.2022.895457

**Published:** 2022-05-09

**Authors:** Alexandru Achim, Tímea Szigethy, Dorottya Olajos, Levente Molnár, Roland Papp, György Bárczi, Kornél Kákonyi, István F. Édes, Dávid Becker, Béla Merkely, Jef Van den Eynde, Zoltán Ruzsa

**Affiliations:** ^1^Division of Invasive Cardiology, Internal Medicine Department, University of Szeged, Szeged, Hungary; ^2^“Niculae Stancioiu” Heart Institute, University of Medicine and Pharmacy “Iuliu Hatieganu”, Cluj-Napoca, Romania; ^3^Cardiac and Vascular Center, Semmelweis University, Budapest, Hungary; ^4^Bács-Kiskun County Hospital, Teaching Hospital of the Szent-Györgyi Albert Medical University, Kecskemét, Hungary; ^5^Department of Cardiovascular Sciences, KU Leuven, Leuven, Belgium

**Keywords:** distal radial access, snuffbox approach, chronic total occlusion, CTO, radiation dose, proximal radial access, radial artery occlusion

## Abstract

**Background:**

Distal radial access (DRA) was recently introduced in the hopes of improving patient comfort by allowing the hand to rest in a more ergonomic position throughout percutaneous coronary interventions (PCI), and potentially to further reduce the rate of complications (mainly radial artery occlusion, [RAO]). Its safety and feasibility in chronic total occlusion (CTO) PCI have not been thoroughly explored, although the role of DRA could be even more valuable in these procedures.

**Methods:**

From 2016 to 2021, all patients who underwent CTO PCI in 3 Hungarian centers were included, divided into 2 groups: one receiving proximal radial access (PRA) and another DRA. The primary endpoints were the procedural and clinical success and vascular access-related complications. The secondary endpoints were major adverse cardiac and cerebrovascular events (MACCE) and procedural characteristics (volume of contrast, fluoroscopy time, radiation dose, procedure time, hospitalization time).

**Results:**

A total of 337 consecutive patients (mean age 64.6 ± 9.92 years, 72.4% male) were enrolled (PRA = 257, DRA = 80). When compared with DRA, the PRA group had a higher prevalence of smoking (53.8% vs. 25.7%, SMD = 0.643), family history of cardiovascular disease (35.0% vs. 15.2%, SMD = 0.553), and dyslipidemia (95.0% vs. 72.8%, SMD = 0.500). The complexity of the CTOs was slightly higher in the DRA group, with higher degrees of calcification and tortuosity (both SMD >0.250), more bifurcation lesions (45.0% vs. 13.2%, SMD = 0.938), more blunt entries (67.5% vs. 47.1%, SMD = 0.409). Contrast volumes (median 120 ml vs. 146 ml, *p* = 0.045) and dose area product (median 928 mGy×cm^2^ vs. 1,300 mGy×cm^2^, *p* < 0.001) were lower in the DRA group. Numerically, local vascular complications were more common in the PRA group, although these did not meet statistical significance (RAO: 2.72% vs. 1.25%, *p* = 0.450; large hematoma: 0.72% vs. 0%, *p* = 1.000). Hospitalization duration was similar (2.5 vs. 3.0 days, *p* = 0.4). The procedural and clinical success rates were comparable through DRA vs. PRA (*p* = 0.6), moreover, the 12-months rate of MACCE was similar across the 2 groups (9.09% vs. 18.2%, *p* = 0.35).

**Conclusion:**

Using DRA for complex CTO interventions is safe, feasible, lowers radiation dose and makes dual radial access more achievable. At the same time, there was no signal of increased risk of periprocedural or long-term adverse outcomes.

## Introduction

Distal radial access (DRA), a technique that can no longer be called “novel” in terms of its widespread adoption, has already been declared feasible and safe in various types of coronary, structural and peripheral procedures (1–7). The most notable advantages are the low rate of radial artery occlusion, few local complications, short hemostasis time and better ergonomics, both for the patient and for the operator (1, 2), especially in the case of left radial artery access.

In recent times, coronary chronic total occlusion (CTO) percutaneous coronary intervention (PCI) has become widely adapted and is currently being performed at large scale, with a significant positive clinical impact on malignant ischemic arrhythmias and adverse clinical outcomes in patients with acute myocardial infarction and incomplete revascularization (8–10). Dual arterial access is necessary in almost every case. Furthermore, these procedures are usually long and arduous. For these reasons, adopting dual DRA and bringing both hands in a physiological position of pronation in close proximity to each other, seems an attractive option. Nonetheless, compared to other well-studied interventions, knowledge about the safety and feasibility of DRA in CTO PCI remains limited (11). The present multicenter, retrospective study aimed to perform a head-to-head comparison between proximal radial access (PRA) and DRA in CTO PCI. We specifically assessed the impact of access strategy on vascular complications, procedural times, and irradiation exposure, provided that procedural efficacy and outcomes remained non-inferior.

## Methods

### Study Patients

All consecutive patients who underwent CTO PCI between May 2016 and October 2021 in 3 Hungarian institutions were included. Because our local protocol has been changed in 2019, switching from PRA to DRA, 2 cohorts could be formed retrospectively, the PRA group (*n* = 257) and the DRA group (*n* = 80). We collected deidentified data of all patients in whom at least one arterial access was either PRA or DRA, in a standardized form. The indication for CTO PCI was established by the local heart team, as well as the recanalization strategy. There were 3 main operators responsible for all the procedures, the learning curve of the DRA as well as its technique being described elsewhere (2). Patients with ultrasound evidence of arterial occlusion, severe calcification, and a lumen of <1 mm were excluded. Baseline patient characteristics, procedural details, puncture-related complications, CTO-related complications, major events at 30 days and 12 months were all recorded in a common database. Before discharge, the patency of the radial artery was verified by duplex ultrasound. After discharge, patients were followed-up by outpatient visits or phone call at 1, 6 and 12 months after the procedure. Written informed consent was obtained from all patients, and the Institution's Ethics Committee approved the study.

### Endpoints

Because our study was a vascular access-related study, focused on the safety, feasibility and performance of DRA in PCI CTO, 2 types of endpoints were defined. The primary outcomes of the study were the success (procedural plus clinical) and access site complications (severe arterial spasm, forearm hematoma, radial artery occlusion, bleeding, pseudoaneurysms and fistulae). The secondary endpoints included were major adverse cardiac and cerebrovascular events (MACCE) and procedural performance characteristics (volume of contrast, fluoroscopy time, radiation dose, procedure time, hospitalization time).

The total procedure time referred to the time interval between the administration of the local anesthetic until the completion of the procedure. For the classification of the forearm hematomas, we used a modified version of the EASY (Early Discharge After Transradial Stenting of Coronary Arteries Study) classification (12). Large hematomas were considered ≥EASY II. Bleeding was considered significant if Bleeding Academic Research Consortium ≥2.

The components of MACCE were defined as non-fatal myocardial infarction (MI), acute stent thrombosis, target lesion revascularization (TLR), stroke or transient ischemic attack, and cardiovascular mortality.

### Statistical Analyses

For the entire cohort (“Before Matching”), continuous variables were evaluated for normality using the Shapiro-Wilk test and reported as mean ± standard deviation or median (interquartile range), as appropriate, while categorical variables were reported as frequencies and percentages. Patients were stratified by approach (PRA vs. DRA) and compared using parametric (Student's paired t) or non-parametric (Mann-Whitney U) tests, as appropriate, for continuous variables and the Chi-squared test for categorical variables.

Propensity score matching was used to adjust for pre-specified baseline characteristics that were potentially confounding variables. We calculated propensity scores using logistic regression models with all baseline variables listed in Table 1, including patient comorbidities and lesion characteristics. The C-statistic for the model was 0.92. PRA cases were matched 1:1 with DRA cases, using the propensity score with a caliper of 0.1 of the standard deviation of the logit of the propensity score, without replacement (13, 14). Standardized mean differences (SMD) were determined to compare baseline characteristics of all patients; a standardized mean difference <0.25 was considered an indicator of good balance between groups (15).

**Table 1:** Baseline characteristics before and after propensity score matching.

**Variable**	**Before matching**				**After matching**			
	**DRA (*n* = 80)**	**PRA (*n* = 257)**	***P*-value**	SMD^*^	**DRA (*n* = 44)**	**PRA (*n* = 44)**	***P*-value**	SMD^*^
Age, years	64.1 (9.58)	64.7 (10.0)	0.623	0.061	62.6 (8.76)	64.6 (10.6)	0.326	0.204
Male sex, *n* (%)	52 (65.0%)	192 (74.7%)	0.120	0.223	32 (72.7%)	27 (61.4%)	0.364	−0.261
BMI, kg/m^2^	29.2 (26.5; 32.3)	29.4 (26.0; 32.3)	0.996	0.005	29.8 (4.80)	29.3 (4.41)	0.586	−0.109
CKD, *n* (%)	16 (20.0%)	34 (13.2%)	0.191	−0.200	12 (27.3%)	10 (22.7%)	0.806	−0.134
Diabetes, *n* (%)	39 (48.8%)	106 (41.2%)	0.292	−0.153	21 (47.7%)	24 (54.5%)	0.670	0.139
AHT, *n* (%)	75 (93.8%)	230 (89.5%)	0.360	−0.139	41 (93.2%)	41 (93.2%)	1.000	0.000
Smoking, *n* (%)	43 (53.8%)	66 (25.7%)	<0.001	−0.643	22 (50.0%)	19 (43.2%)	0.669	−0.156
Family history of CVD, *n* (%)	28 (35.0%)	39 (15.2%)	<0.001	−0.553	14 (31.8%)	10 (22.7%)	0.473	−0.253
Dyslipidemia, *n* (%)	76 (95.0%)	187 (72.8%)	<0.001	−0.500	40 (90.9%)	40 (90.9%)	1.000	0.000
Previous MI, *n* (%)	35 (43.8%)	115 (44.7%)	0.978	0.020	21 (47.7%)	21 (47.7%)	1.000	0.000
Previous CABG, *n* (%)	8 (10.0%)	35 (13.6%)	0.512	0.106	6 (13.6%)	6 (13.6%)	1.000	0.000
PAD, *n* (%)	23 (28.7%)	58 (22.6%)	0.327	−0.148	10 (22.7%)	8 (18.2%)	0.792	−0.109
Diagnosis			0.096				0.761	
Cx, *n* (%)	7 (8.75%)	49 (19.1%)		0.263	6 (13.6%)	8 (18.2%)		0.116
LAD, *n* (%)	31 (38.8%)	88 (34.2%)		−0.095	17 (38.6%)	18 (40.9%)		0.048
RCA, *n* (%)	42 (52.5%)	120 (46.7%)		−0.116	21 (47.7%)	18 (40.9%)		−0.137
Location			0.454				1.000	
Distal, *n* (%)	5 (6.25%)	15 (5.84%)		−0.018	0 (0.00%)	1 (2.27%)		0.097
Mid, *n* (%)	24 (30.0%)	97 (37.7%)		0.160	17 (38.6%)	17 (38.6%)		0.000
Proximal, *n* (%)	51 (63.7%)	145 (56.4%)		−0.148	27 (61.4%)	26 (59.1%)		−0.046
Lesion length, mm	30.0 (20.0; 40.0)	25.0 (20.0; 40.0)	0.076	−0.110	35.0 (25.0; 40.0)	30.0 (25.0; 42.5)	0.859	0.176
Lumen diameter, mm	3.00 (2.50; 3.50)	2.75 (2.50; 3.00)	0.001	−0.544	3.00 (2.50; 3.00)	2.88 (2.50; 3.00)	0.655	−0.033
Calcification			<0.001				1.000	
Extreme, *n* (%)	30 (37.5%)	43 (16.7%)		−0.556	15 (34.1%)	16 (36.4%)		0.061
Severe, *n* (%)	24 (30.0%)	119 (46.3%)		0.327	11 (25.0%)	11 (25.0%)		0.000
Slight, *n* (%)	20 (25.0%)	87 (33.9%)		0.187	14 (31.8%)	14 (31.8%)		0.000
No, *n* (%)	6 (7.50%)	8 (3.11%)		−0.253	4 (9.09%)	3 (6.82%)		−0.131
Tortuosity			<0.001				0.890	
Extreme, *n* (%)	5 (6.25%)	7 (2.72%)		−0.217	2 (4.55%)	2 (4.55%)		0.000
Severe, *n* (%)	21 (26.2%)	23 (8.95%)		−0.606	8 (18.2%)	11 (25.0%)		0.239
Slight, *n* (%)	34 (42.5%)	198 (77.0%)		0.821	23 (52.3%)	20 (45.5%)		−0.162
No, *n* (%)	20 (25.0%)	29 (11.3%)		−0.434	11 (25.0%)	11 (25.0%)		0.000
Bifurcation, *n* (%)	36 (45.0%)	34 (13.2%)	<0.001	−0.938	14 (31.8%)	13 (29.5%)	1.000	−0.067
JCTO score			0.080	−0.294			0.433	−0.059
0, *n* (%)	0 (0.00%)	4 (1.56%)			0 (0.00%)	0 (0.00%)		
1, *n* (%)	8 (10.0%)	30 (11.7%)			3 (6.82%)	3 (6.82%)		
2, *n* (%)	37 (46.2%)	134 (52.1%)			20 (45.5%)	18 (40.9%)		
3, *n* (%)	25 (31.2%)	79 (30.7%)			15 (34.1%)	21 (47.7%)		
4, *n* (%)	10 (12.5%)	10 (3.89%)			6 (13.6%)	2 (4.55%)		
Blunt entry shape, *n* (%)	54 (67.5%)	121 (47.1%)	0.002	−0.409	26 (59.1%)	27 (61.4%)	1.000	0.046
Occlusion length >20 mm, *n* (%)	61 (76.2%)	179 (69.6%)	0.319	−0.144	37 (84.1%)	40 (90.9%)	0.519	0.148
Right coronary dominance, *n* (%)	76 (95.0%)	228 (88.7%)	0.151	−0.199	41 (93.2%)	40 (90.9%)	1.000	−0.072

*AHT, arterial hypertension; BMI, body mass index; CABG, coronary artery bypass grafting; CKD, chronic kidney disease; CVD, cerebrovascular disease; Cx, circumflex coronary artery; DRA, distal radial access; LAD, left anterior descending coronary artery; MI, myocardial infarction; PAD, peripheral artery disease; PRA, proximal radial access; RCA, right coronary artery; SMD, standardized mean difference*.

* *PRA minus DRA*.

For the matched cohort (“After Matching”), data were again presented as described above. Both approaches (PRA vs. DRA) were compared by paired univariate analysis. Categorical variables were compared using McNemar's test and continuous variables were compared by Wilcoxon signed rank test. All analyses were completed with R Statistical Software (version 4.1.1, Foundation for Statistical Computing, Vienna, Austria).

## Results

### Study Population

**Figure 1:**
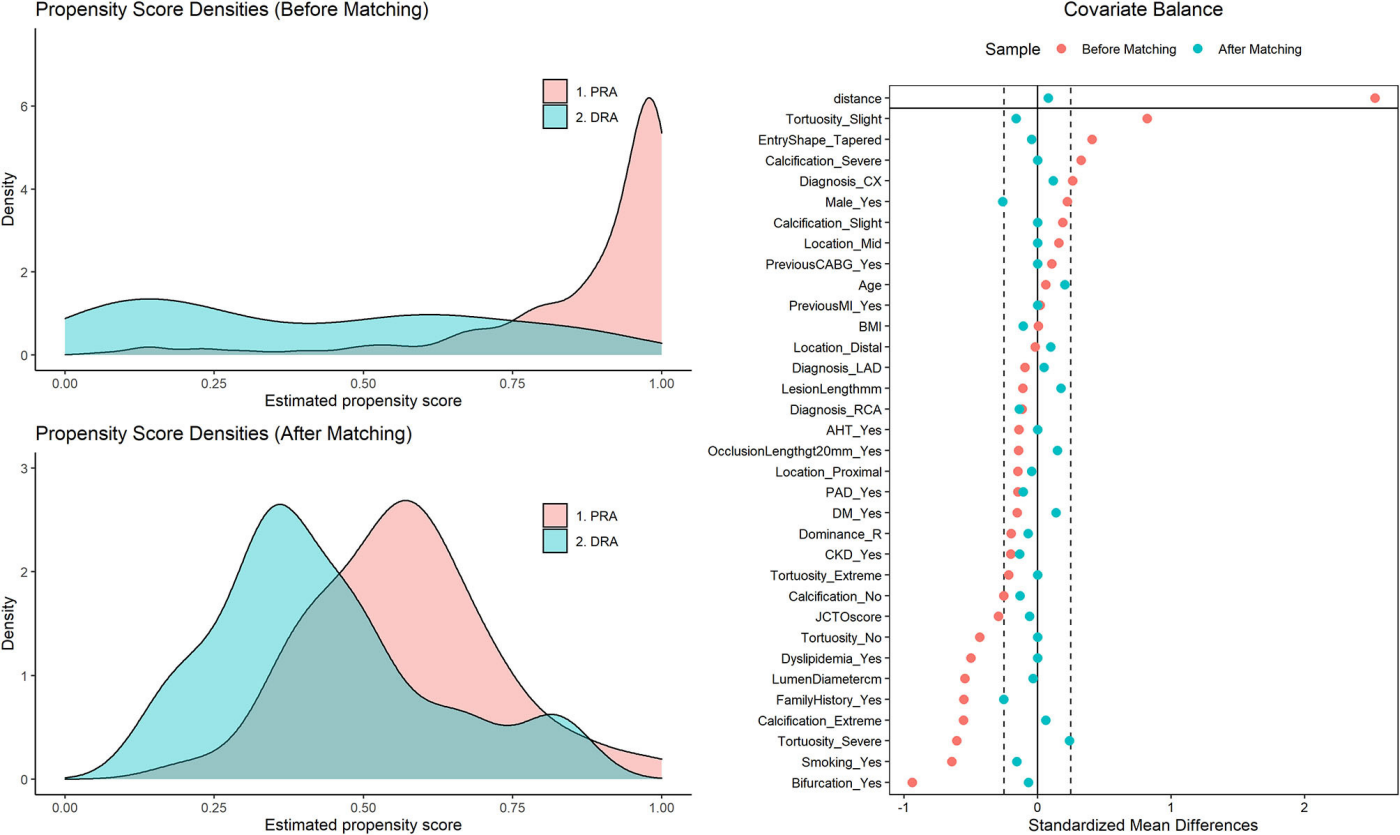
Validation of propensity score matching. (Left) Density of propensity scores for cases in the PRA and the DRA group before and after matching. The propensity scores represent the probability for each patient of belonging to the PRA group. The overlapping area represents patients with similar propensity scores available for close matches. (Right) “Love Plot” illustrating the covariate balance created in the propensity score matched sample. The standardized mean differences comparing covariates between the PRA and DRA groups are shown both in the original sample and after propensity score matching. While there were relevant differences (>25%) in covariates between both groups in the original sample, after matching all are <25%, indicating balance between cases in the PRA and DRA groups for all relevant covariates. DRA, distal radial access; PRA, proximal radial access.

A total of 337 consecutive patients (mean age 64.6 ± 9.92 years, 72.4% male) underwent PCI between May 2016 and October 2021 at our institutions. Of these, access was obtained using PRA in 257 and using DRA in 80 cases. Baseline characteristics of the unmatched cohort are presented in Table 1. When compared with DRA, the PRA group had a higher prevalence of smoking (53.8% vs. 25.7%, SMD = 0.643), family history of cardiovascular disease (35.0% vs. 15.2%, SMD = 0.553), and dyslipidemia (95.0% vs. 72.8%, SMD = 0.500). The prevalence of the other risk factors was similar. The complexity of the CTOs was slightly higher in the DRA group, being characterized by higher degrees of calcification and tortuosity of the target lesions (both SMD > 0.250), and a higher prevalence of bifurcation lesions (45.0% vs. 13.2%, SMD = 0.938) and blunt entry shape (67.5% vs. 47.1%, SMD = 0.409). Propensity score matching resulted in 44 pairs, which showed adequate overall balancing in the baseline characteristics (SMD < 25%), except for very minor residual imbalances in male sex and family history of cardiovascular disease (SMD for PRA compared to DRA of −0.261 and −0.253, respectively) (Table 1, Figure 1).

### Intraprocedural Characteristics

Intraprocedural characteristics are summarized in Table 2. In the unmatched cohort, the distribution of CTO was significantly different between both groups: anterograde dissection reentry was more frequent in the DRA group (27.8% vs. 3.89%, *p* < 0.001) whereas anterograde wire escalation, retrograde dissection reentry, and retrograde wire escalation were more frequent in the PRA group (all *p* < 0.001). Cases in the DRA group had higher use of intravascular ultrasound (IVUS, 16.2% vs. 7.39%, *p* = 0.032), greater use of guidewires (median 3.00 vs. 2.00, *p* = 0.001), and longer stents (median 56.5 mm vs. 40.0 mm, *p* < 0.001). Furthermore, contrast volumes (median 120 ml vs. 146 ml, *p* = 0.045) and dose area product (DAP) (median 928 mGy × cm^2^ vs. 1,300 mGy × cm^2^, *p* < 0.001) were lower in the DRA group. On the other hand, PRA was characterized by shorter procedure times (median 38.5 min vs. 55.0 min, *p* < 0.001) and fluoroscopy times (median 19.0 vs. 27.5 min, *p* = 0.042).

**Table 2:** Intraprocedural characteristics.

**Variable**	**Before matching**			**After matching**		
	**DRA (*n =* 80)**	**PRA (*n =* 257)**	***P*-value**	**DRA (*n =* 44)**	**PRA (*n =* 44)**	***P*-value**
CTO technique			<0.001			0.001
Anterograde dissection reentry, *n* (%)	22 (27.8%)	10 (3.89%)		15 (34.9%)	3 (6.82%)	
Anterograde wire escalation, *n* (%)	55 (69.6%)	218 (84.8%)		26 (60.5%)	36 (81.8%)	
Retrograde dissection reentry, *n* (%)	1 (1.27%)	10 (3.89%)		1 (2.33%)	0 (0.00%)	
Retrograde wire escalation, *n* (%)	1 (1.27%)	19 (7.39%)		1 (2.33%)	5 (11.4%)	
Rotational atherectomy, *n* (%)	9 (11.2%)	23 (8.98%)	0.701	3 (6.82%)	5 (11.6%)	0.484
Dual access, *n* (%)	46 (57.5%)	130 (50.6%)	0.340	26 (59.1%)	25 (56.8%)	1.000
Antegrade approach used, *n* (%)	78 (97.5%)	252 (98.1%)	0.672	42 (95.5%)	44 (100%)	0.494
Retrograde approach used, *n* (%)	6 (7.50%)	13 (5.06%)	0.410	6 (13.6%)	2 (4.55%)	0.266
IVUS, *n* (%)	13 (16.2%)	19 (7.39%)	0.032	7 (15.9%)	2 (4.55%)	0.157
Number of guidewires	3.00 (2.00; 5.00)	2.00 (1.00; 4.00)	0.001	3.00 (2.00; 6.00)	2.50 (1.75; 3.00)	0.003
Number of balloons	3.00 (2.00; 4.00)	3.00 (2.00; 4.00)	0.431	3.00 (2.00; 4.25)	3.00 (2.00; 4.00)	0.268
Stent length, mm	56.5 (37.5; 82.0)	40.0 (22.0; 64.0)	<0.001	59.0 (41.5; 79.8)	46.0 (28.0; 68.8)	0.071
Contrast volume, ml	120 (90.0; 190)	146 (100; 218)	0.045	142 (100; 205)	157 (119; 212)	0.447
Procedure time, min	55.0 (33.8; 87.0)	38.5 (20.0; 64.0)	<0.001	70.0 (40.0; 104)	27.5 (15.0; 69.2)	<0.001
DAP, mGy × cm^2^	928 (400; 1500)	1,300 (593; 2787)	<0.001	1,000 (445; 1500)	1,515 (668; 3097)	0.018
Fluoroscopy time, min	27.5 (10.0; 52.0)	19.0 (10.0; 31.0)	0.042	34.5 (13.0; 61.2)	21.5 (14.8; 32.8)	0.064

*CTO, chronic total obstruction; DRA, distal radial access; IVUS, intravascular ultrasound; PRA, proximal radial access; DAP, dose area product*.

After matching, anterograde dissection reentry was still more frequent (34.9% vs. 6.82%, *p* = 0.001) and the number of guidewires used was still higher (median 3.00 vs. 2.50, *p* = 0.003) in DRA than in PRA. Lower DAP (median 1,000 mGy × cm^2^ vs. 1,515 mGy × cm^2^, *p* = 0.018) and longer procedure time (median 70.0 min vs. 37.5 min, *p* < 0.001) were also still observed for the DRA group. There was still a trend toward longer stent length (*p* = 0.071) and longer fluoroscopy time (*p* = 0.064) in the DRA group, although this did not reach statistical significance.

**Figure 2:**
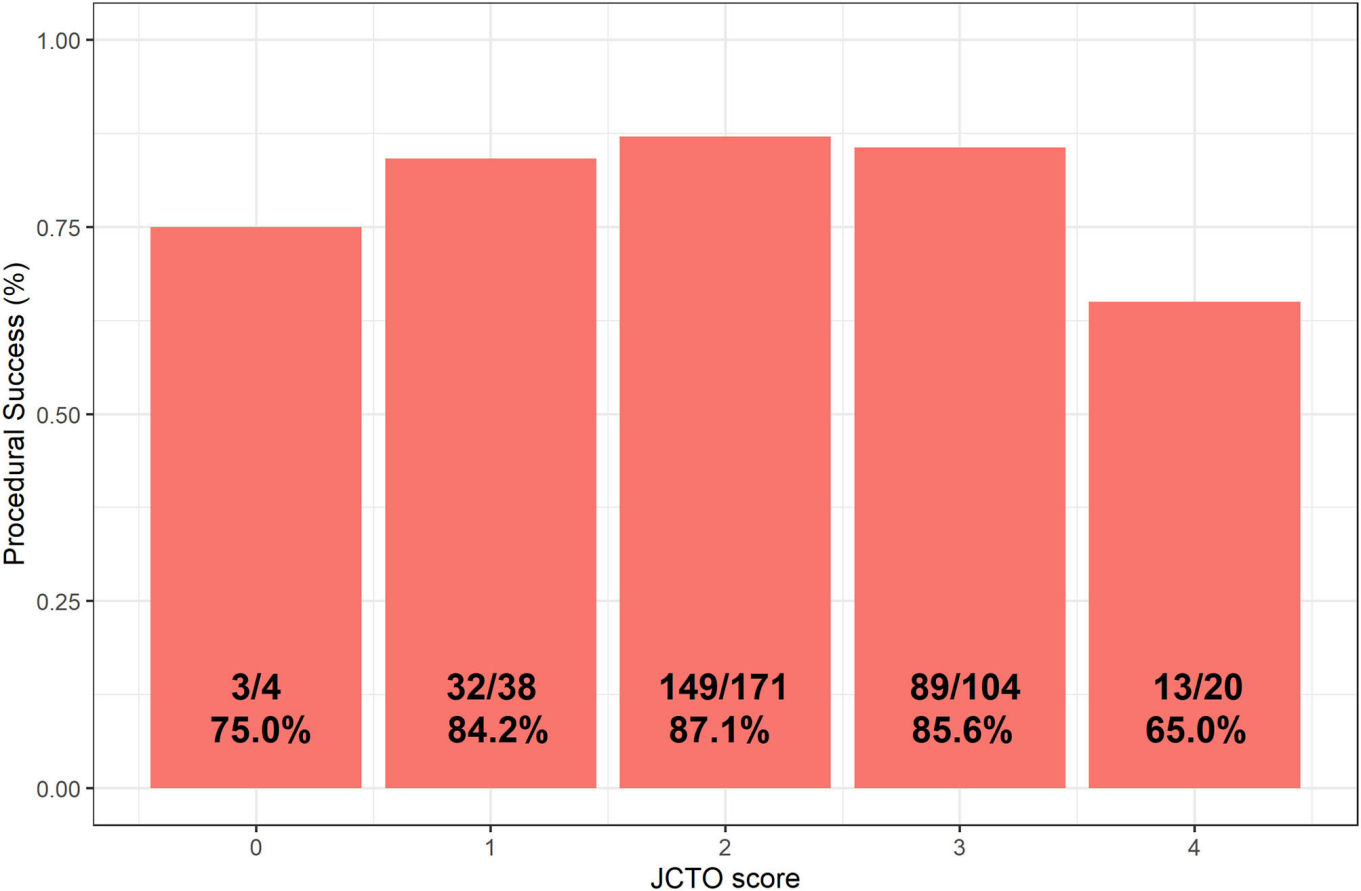
Distribution chart showing procedural CTO success as a function of JCTO score. CTO, chronic total obstruction.

The overall complexity of the procedures remained varied across all patients, although most had a Japanese chronic total occlusion (JCTO) score ≤2 (*n* = 171). However, no clear correlation between JCTO score and procedural success could be established (Figure 2).

### Procedural and Long-Term Outcomes

Procedural and long-term outcomes are presented in Table 3. In the unmatched cohort, a shorter hospital length of stay (median 2.00 days vs. 3.00 days, *p* = 0.006) was observed in the DRA group. Furthermore, the 12-months rate of MACCE tended to be lower in the DRA group (10.0% vs. 20.2%, *p* = 0.055), although this did not reach statistical significance. Numerically, local vascular complications were more common in the PRA group, although these did not meet statistical significance (radial artery occlusion [RAO]: 2.72% vs. 1.25%, *p* = 0.450; large hematoma: 0.72% vs. 0%, *p* = 1.000). After matching, no differences were observed in any of the observed outcomes.

**Table 3:** Procedural and long-term outcomes.

**Variable**	**Before matching**			**After matching**		
	**DRA (*n* = 80)**	**PRA (*n* = 257)**	***P*-value**	**DRA (*n* = 44)**	**PRA (*n* = 44)**	***P*-value**
**Procedural outcomes**						
Access site complications			0.820			1.000
Large hematoma, *n* (%)	0 (0.00%)	2 (0.78%)	1.000	0 (0.00%)	0 (0.00%)	1.000
Small hematoma, *n* (%)	2 (2.50%)	4 (1.56%)	0.577	2 (4.55%)	1 (2.27%)	0.557
RAO, *n* (%)	1 (1.25%)	7 (2.72%)	0.450	0 (0.00%)	1 (2.27%)	1.000
Bleeding, *n* (%)	0 (0.00%)	0 (0.00%)	1.000	0 (0.00%)	0 (0.00%)	1.000
None, *n* (%)	77 (96.2%)	244 (94.9%)	0.631	42 (95.5%)	42 (95.5%)	1.000
Any complications^*^, *n* (%)	7 (8.75%)	10 (3.89%)	0.138	3 (6.82%)	3 (6.82%)	1.000
Procedural success, *n* (%)	73 (91.2%)	213 (82.9%)	0.100	38 (86.4%)	39 (88.6%)	1.000
Clinical success, *n* (%)	70 (87.5%)	167 (79.5%)	0.161	37 (84.1%)	32 (78.0%)	0.664
Hospital length of stay, days	2.00 (2.00; 3.00)	3.00 (2.00; 4.00)	0.006	2.50 (2.00; 3.25)	3.00 (2.00; 3.25)	0.412
**Long-term outcomes**						
30-day MACCE	3 (3.75%)	11 (4.28%)	1.000	2 (4.55%)	2 (4.55%)	1.000
6-months MACCE	7 (8.75%)	31 (12.1%)	0.538	4 (9.09%)	4 (9.09%)	1.000
12-months MACCE	8 (10.0%)	52 (20.2%)	0.055	4 (9.09%)	8 (18.2%)	0.351
12-months redo PCI	6 (7.50%)	27 (10.5%)	0.566	5 (11.4%)	5 (11.4%)	1.000
12-months target lesion revascularization	3 (3.75%)	12 (4.67%)	1.000	1 (2.27%)	4 (9.09%)	0.360
12-months stent thrombosis	1 (1.25%)	1 (0.39%)	0.419	1 (2.27%)	0 (0.00%)	1.000
12-months MI	2 (2.50%)	3 (1.17%)	0.340	1 (2.27%)	0 (0.00%)	1.000
12-months TIA or stroke	2 (2.50%)	2 (0.78%)	0.240	1 (2.27%)	0 (0.00%)	1.000
12-months death	0 (0.00%)	9 (3.50%)	0.122	44 (100%)	44 (100%)	1.000

*DRA, distal radial access; RAO, radial artery occlusion; MACE, major adverse cardiac events; MI, myocardial infarction; PCI, percutaneous coronary intervention; PRA, proximal radial access; TIA, transient ischemic attack*.

* *These included cardiac decompensation, coronary dissection, coronary perforation, and pericardial fluid/tamponade*.

## Discussion

The main findings of our study were that (1) procedure success rates, complication rates, and long-term outcomes were comparable after CTO recanalization through DRA vs. PRA; and (2) despite longer procedure times, DRA was associated with lower radiation doses. These findings suggest that DRA may be an attractive and ergonomic alternative to PRA that is as safe and effective for CTO procedures. Moreover, although not statistically significant, the RAO rate seems to be lower with DRA, which is of clinical importance because, for many patients, this is not their last intervention in the catheterization room.

Only 2 previous studies tested the feasibility of DRA in CTO recanalization procedures, but none had a PRA control group or quantified the radiation dose (11, 16). In a small, prospective, multicenter study (41 patients), Gasparini et al. (16) demonstrated high procedural success (90.3%) using the 7-French Glidesheath Slender for CTO PCIs through left DRA only, their operators using ultrasound-guided puncture as well. Vascular access-site complications (DRA-related) or MACEs were not recorded. The cohort of Lin et al. (7) was larger (298 patients) and, also often used the Glidesheath system in the majority of their patients (95.5%). The investigators observed low vascular complications rates (RAO 0.5%, large hematomas 0.2%), consistent with our data and those by Gasparini et al. (16). Interestingly, they reported that successful DRA was feasible even in 2 cases (0.7%) with prior pre-existing RAO at the ipsilateral side, by resolving the ROA by means of angioplasty first.

**Figure 3:**
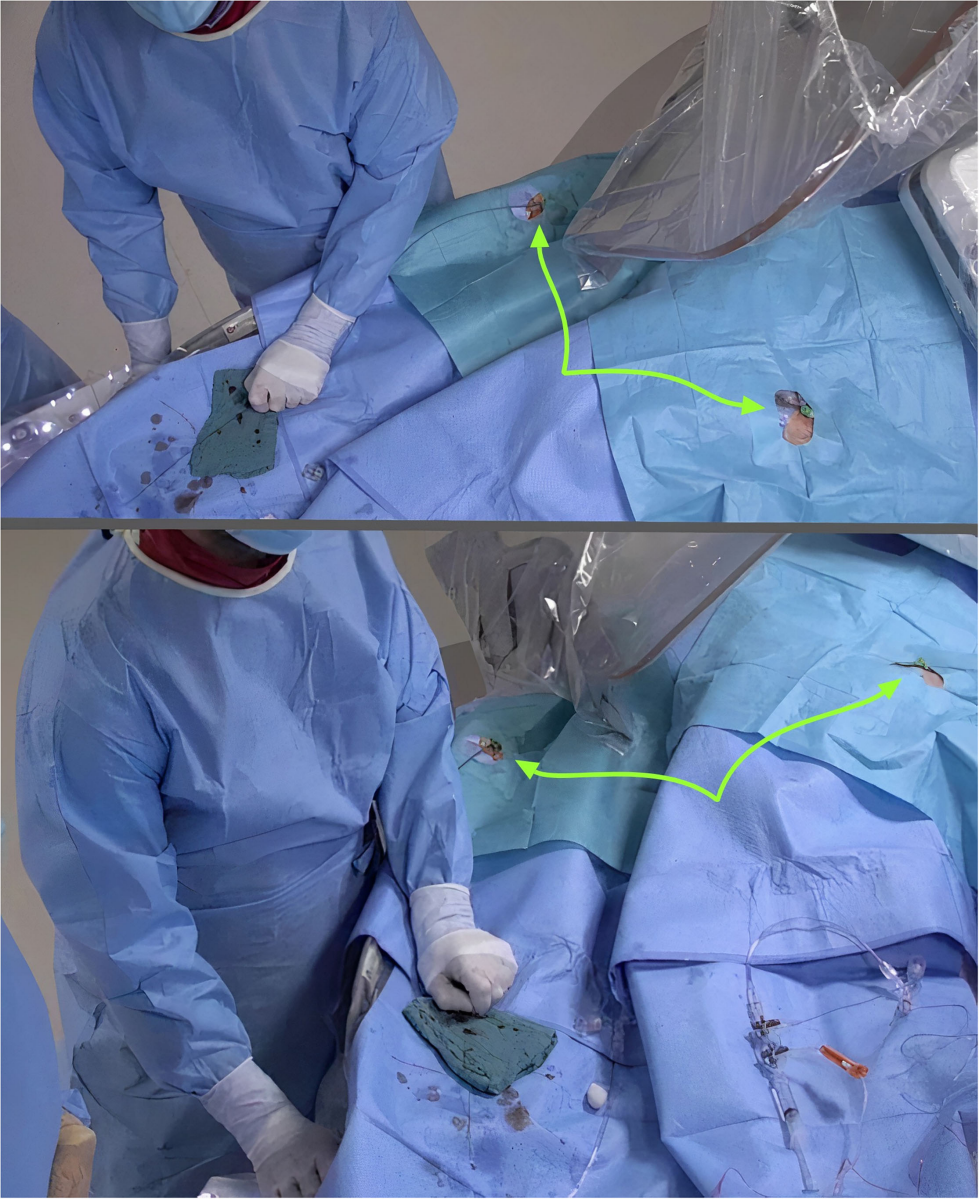
Improved ergonomics during dual distal radial (arrows) in CTO PCI.

Our findings are clinically important for several reasons. First, as mentioned earlier, CTO PCI often requires dual arterial access. From the operator's point of view, it is ergonomically easier when using the left radial artery; the arm can be then positioned over the patient's right groin without the need of maintaining a supine position, rather than having to bend over the patient which can become wearisome during long procedures in obese patients. At the same time, it allows a safer distance between the operator and the radiation source. The use of DRA is also more comfortable for the patient as the arm can be put in a neutral position without wrist rotation and no extra support devices are required in cases of left forearm use (Figure 3). This may be proven important for patients with orthopedic problems, including frozen shoulders (17). The hemostasis time is shorter in comparison to traditional radial approach as the artery at this level has a smaller diameter and is easily compressible. Furthermore, the patient is able to bend the wrist with no restriction after the procedure, thus making it better tolerated. In a similar population, patients have reported a higher rate of satisfaction post recovery after DRA use in comparison to the conventional radial access (18).

Second, in terms of radioprotection, the lower DAP with DRA in our study (a 34% reduction compared to PRA in matched analyses) is encouraging. In this regard, DRA may help to effectively address one of the main disadvantages of traditional radial access compared to transfemoral access, i.e., greater radiation exposure (19, 20). this imbalance could be equated with DRA. Ultimately, bringing both hands over the patient's pelvis is equivalent to the transfemoral positioning (2).

Third, although statistical significance could not be determined given sample size limitations, the current study suggested a 1.47–2.27% absolute risk reduction of RAO with DRA. Several mechanisms, including vascular injury, blood flow reduction, and thrombosis, have been linked to the occurrence of RAO (21). The 2019 international consensus paper on “Best Practices for the Prevention of Radial Artery Occlusion After Transradial Diagnostic Angiography and Intervention” recommends a 5% RAO rate threshold and proposes DRA as a potential approach to avoid RAO given its anatomic basis and physiological rationale (22). Notably, both groups in our study fell below this limit. Moreover, in the context of using large 7-French sheaths in CTO PCI, the risk of RAO has still dropped. Of course, operator's expertise is equally important to improve procedural success and diminish crossover rate among patients undergoing DRA. Issues such as the appropriate choice of sheath and catheter sizes to minimize arterial wall injury, adequate procedural anticoagulation, non-occlusive techniques, and adequate hemostasis (e.g., “patent hemostasis”) are important steps to further improve this technique (23). In one of our previous reports, we found that it takes at least 150 cases to reach the learning curve and maintain a consistently high success rate of >94.0% (2). These findings are also consistent with a Korean report (24).

All the above mentioned aspects have meaningful implications in the CTO procedures. Maintaining a radial-only procedure, efficiently using 7-French catheters, and working comfortably away from the radiation source indeed represent important advances for the CTO community. Nonetheless, appropriate size matching between the catheter and the radial artery diameter may theoretically introduce another challenge when pursuing DRA. In a large registry of over 1,000 patients, the mean diameter in the distal segment was of 2.3 ± 0.5 mm, while the outer diameter of the 7-French Glidesheath is 2.79 mm, and that of the 6-F is 2.46 mm (2). Another study found even smaller diameters (2.01 ± 0.53 mm, 19% smaller than the proximal segment) (25). Nonetheless, none of our patients required crossover due to severe arterial spasm and only one patient developed distal RAO (1.25%). It should be noted, however, that our internal protocol specifies that the DRA should be punctured under guidance of duplex ultrasound. The planning of the procedure by means of pre- and peri-procedural ultrasound certainly helped in this regard. Thus, our data provide further insight into the impressive versatility of the radial artery wall, which can accommodate devices larger than the nominal size regardless of age, body weight and vessel anatomy (7, 26, 27).

Finally, in terms of broader implications, the clinical benefits of DRA over conventional PRA during long-term follow-up are still to be determined. One of the key goals of future research should be to investigate whether this access site may deliver added benefits on “hard” clinical endpoints while maintaining the same efficacy as traditional PRA. Our experience offers a promising first window into these potential benefits.

In terms of the CTO PCI rationale, the authors wish to acknowledge several benefits of such a procedure. It was shown that the presence of a coronary CTO was associated with increased rates of all-cause mortality at midterm follow-up and the composite endpoint of cardiac death at 24 h, recurrent ventricular tachyarrhythmias, and appropriate ICD therapies at 18 months (9). Viable myocardium supplied by a CTO is a persistently ischemic zone (28). Moreover, with respect to complete revascularization, a trend was noted toward better in-hospital/30-day mortality and 6-month health status in patients with a lower residual Syntax Score (8, 10, 29). This is of particular importance when a patient with a coronary CTO suffers an acute MI in the donor vessel (“double jeopardy” effect). However, improving patient symptoms caused by myocardial ischemia (angina, exertional dyspnea, and sometimes fatigue) despite optimal medical therapy remains the only benefit of CTO-PCI that has been demonstrated in randomized, controlled trials and should therefore currently be the primary indication for offering this procedure to patients (30).

There are several limitations of our study that are worthy of mentioning. First, the retrospective nature of our study is subject to confounding; nevertheless, all included patients were consecutive patients and propensity score matching was performed to balance any clinically meaningful confounders between the two groups. Second, a specific protocol for ultrasound-guided puncture and transradial band air removal to target faster hemostasis was introduced for all DRA cases, while this protocol was not employed for conventional PRA. The potential impact of this protocol can thus not entirely be separated from the observed effect of the approach (DRA vs. PRA). Third, the data were only analyzed based on intention-to-treat whilst the rate of DRA failure and crossover percentage were not registered. We know that the lumen of the radial artery is slightly smaller at the anatomical snuffbox and that inserting a sheath can be more challenging, especially in women (31). Therefore, beyond patient discomfort and increased radiation exposure, transradial access crossover may entail delayed revascularization and worse outcomes compared with successful radial access in acute coronary syndrome patients and abolishes the bleeding benefit offered by radial access over femoral access (32, 33). However, in the setting of CTO, this clinical impact is not of such significant importance.

## Conclusion

Using DRA for complex CTO interventions is safe, feasible, lowers radiation dose and makes dual radial access more achievable. At the same time, there was no signal of increased risk of periprocedural or long-term adverse outcomes.

## Data Availability Statement

The raw data supporting the conclusions of this article will be made available by the authors, without undue reservation.

## Ethics Statement

The studies involving human participants were reviewed and approved by Institutional Review Board of Hungarian State Ethical Review (OGYÉI/50275/2018). The patients/participants provided their written informed consent to participate in this study.

## Author Contributions

AA, KK, BM, DB, IÉ, GB, RP, and ZR contributed to the conception and design of the study. AA and JV organized the database. AA wrote the first draft of the manuscript. TS, DO, JV, and ZR wrote sections of the manuscript. All authors contributed to manuscript revision, read, and approved the submitted version.

## Conflict of Interest

The authors declare that the research was conducted in the absence of any commercial or financial relationships that could be construed as a potential conflict of interest.

## Publisher's Note

All claims expressed in this article are solely those of the authors and do not necessarily represent those of their affiliated organizations, or those of the publisher, the editors and the reviewers. Any product that may be evaluated in this article, or claim that may be made by its manufacturer, is not guaranteed or endorsed by the publisher.

## Abbreviations

DRA: distal radial access
CTO: chronic total occlusion
PCI: percutaneous coronary intervention
PRA: proximal radial access
MACCE: major adverse cardiac and cerebrovascular events
MI: myocardial infarction
TLR: target lesion revascularization
SMD: standardized mean difference
DAP: dose area product
RAO: radial artery occlusion
JCTO: Japanese chronic total occlusion.
